# Development and validation of antisnake venom knowledge assessment tool (AKAT) for healthcare practitioners

**DOI:** 10.1016/j.toxcx.2020.100064

**Published:** 2020-12-03

**Authors:** Auwal A. Bala, Abubakar I. Jatau, Ismaeel Yunusa, Mustapha Mohammed, Al-Kassim H. Mohammed, Abubakar M. Isa, Wada A. Sadiq, Kabiru A. Gulma, Inuwa Bello, Sani Malami, Godpower C. Michael, Basheer A.Z. Chedi

**Affiliations:** a Department Pharmacology, College of Medicine and Health Sciences, Federal University Dutse, Nigeria; b Department of Pharmacology and Therapeutics, Bayero University, Kano, Nigeria; c School of Pharmacy and Pharmacology, University of Tasmania, Tasmania, Australia; d University of South Carolina College of Pharmacy, Columbia, South Carolina, USA; e Harvard School of Public Health, Boston, MA, USA; f School of Pharmaceutical Sciences, Universiti Sains Malaysia, 11800 Penang, Malaysia; g Department of Clinical Pharmacy and Pharmacy Practice, Ahmadu Bello University Zaria, Kaduna, Nigeria; h Faculty of Pharmaceutical Sciences, Bayero University Kano, Nigeria; i Malaria Consortium, Jigawa State Office, Nigeria; j School of Global Health and Bioethics, Euclid University, Gambia; k Jigawa State Hospital Services, Dutse, Nigeria; l Department of Family Medicine, Aminu Kano Teaching Hospital, Kano, Nigeria; m Venom-Antivenom Research Project (VASP) and Nigeria- Snakebite Research and Intervention Centre (N-SRIC), Nigeria

**Keywords:** Antisnake venom, Knowledge assessment, Healthcare practitioners, Tool validation

## Abstract

Antisnake venom (ASV) is the only specific and standard treatment for snakebite envenoming worldwide. The knowledge of antivenom dosage, mode of administration, availability, and logistics is essential to the healthcare practitioners (HCPs) in the management of snakebites. It is vital for the HCPs involved in the handling of ASVs to have its basic knowledge. The ASV contains proteins and can, therefore, easily get denatured if not handled appropriately, leading to poor therapeutic outcome. It is also essential for clinicians to be aware of the tendency of ASV to cause a severe life-threatening hypersensitivity reaction. There is currently no validated tool for assessing the knowledge of ASV among HCPs. Therefore, we developed and validated a tool for evaluating the HCPs knowledge of ASV. The items included in the tool were first generated from a comprehensive literature review. Face validity were conducted by presenting the drafted tool to ten experts on the subject matter. A validation study was conducted among doctors, pharmacists, nurses, pharmacy technicians, and the general public. The objectives of the study were to test the tool for content validity using the content validity index (CVI), construct validity using contrast group approach, difficulty index, readability, and reliability test using the test-retest method. We developed and validated a final tool containing thirty-three items. The tool was valid for face validity and had a scale-level (average) content validity (S-CVI/Ave) of 0.91. The ASV knowledge of pharmacists was higher than that of doctors, pharmacy technicians, nurses, and the general public (*p* < 0.001), thus, valid for construct validity. The readability of the tool using the Simple Measure of Gobbledygook (SMOG) was determined to be grade level 7. The test-retest analysis showed no significant difference between the mean knowledge scores measured at four weeks interval (*p* = 0.916), implying excellent reliability. The AKAT has demonstrated good psychometrical properties that would enable its application among a wide range of healthcare practitioners.

## Highlights

• A tool for assessing knowledge of anti-snake venom was developed and validated.
• Psychometrics approach was used in validating the knowledge assessment tool.
• The tool is reliable with a Simple Measure of Gobbledygook grade level 7.
• The tool can measure anti-snake venom knowledge among healthcare practitioners.

## 1 Introduction

Snakebite envenoming (SBE) is a high-priority neglected tropical disease and a significant public health problem in tropical and subtropical regions (WHO, 2017). Snakebite-related mortality is highest in resource-poor countries and is directly related to socioeconomic indicators of poverty (Harrison et al., 2009). The highest burden of morbidity and mortality associated with snakebite is seen in the rural disadvantaged communities of tropical countries in South Asia, Southeast Asia, and sub-Saharan Africa (Cruz et al., 2009). Increased exposure to snakes due to traditional agricultural practices, lack of adequate healthcare services, poor access to available services, and lack of effective antisnake venom contributed to this healthcare burden (Kasturiratne et al., 2008; Chippaux, 2008).

The knowledge of antisnake venom (ASV), its dosage, mode of administration, availability, and logistics (transportation and storage) is vital to healthcare practitioners (HCPs), particularly those that are involved in its handling (WHO 2017). The efforts and advocacy for the availability of more ASV will be in vain if not appropriately handled before utilized by end-users. This concern probably informed the choice of one of the goals of the International Society on Toxinology (IST) that includes promotion of research initiatives to improve epidemiological and clinical knowledge of envenoming and to enhance the training of HCPs on antivenom usage and quality control (Gutiérrez et al., 2017).

Several tools have been used to assess first aid and general knowledge of snakebite envenoming in Asia and Africa, including Hong Kong (Fung et al., 2009), West Bengali (Das et al., 2015), Nepal (Subedi et al., 2018), Savannakhet Province of Laos (Inthanomchanh et al., 2017) and Cameroon (Tabei et al., 2010). These tools were majorly used in assessing baseline knowledge among doctors, nurses, and medical students. In a recent study in Nigeria, Michael et al. (2018) used a tool in evaluating the general knowledge of snakebite envenoming among doctors in Northern Nigeria. However, based on our findings, there were no previous studies that revealed detailed psychometric tests in the development of the ASV knowledge assessment tool. Therefore, it is essential to develop a tool that will assess ASV knowledge among HCPs that can be used by all stakeholders in the healthcare sector.

### 1.1 Rationale and aim of the study

Antisnake venoms are purified antibodies against venom, can cause life-threatening hypersensitivity reactions, and easily denatured when handled inappropriately. Experts have suggested that HCPs in charge of the management of SBE should be knowledgeable in the fundamental aspects of its therapy as well as the nature and logistics requirements of ASV (Gutiérrez et al., 2009). Inadequate knowledge and skills of ASV among HCPs in Nigeria is an issue that requires modalities and prompt interventions to improve their knowledge. Moreover, training was found to significantly improve knowledge of snakebite management among HCPs in Cameroon (Taieb et al., 2018). We, therefore, provide a validated and reliable tool that can be used in assessing HCPs' knowledge of ASV.

## 2 Methods

The tool development and validation methods were based on the guidelines for developing and validating questionnaires (Tsang et al., 2017).

### 2.1 Development

Items included in the draft tool were generated from a review of literature and discussion with experts on the subject matter. A search of studies was performed using Scopus, Embase via Ovid, and Medline via PubMed, from April to December 2019. Items were generated from studies on ASVs identified from the review. The items in the draft tool developed were designed to measure a single construct (ASV knowledge). They covered six domains, namely ASV definition, dosage, mode of administration, availability, cost, and logistics. The tool also contains items related to socio-demographic characteristics. Thus, the tool was designed to contain three sections: (a) socio-demographic characteristics, (b) ASV definitions, dosage, mode of administration and source of ASV knowledge, and (c) ASV availability, cost, and storage and handling requirements.

The tool was semi-structured, containing both open and closed-ended questions. Several “detractors” were added to identify unguarded responses by the participants. The items were concise and straightforward for easy comprehension and self-administration.

### 2.2 Tool validation

#### 2.2.1 Face validity

To ensure face validity, we presented the draft tool to a panel of experts consisting of ten independent HCPs with clinical and research knowledge of ASV. The panel members consist of doctors and pharmacists. The doctors are members of the Nigeria-Snakebite Research and Intervention Centre (N-SRIC). They are medical specialists in infectious disease and tropical medicine with research experience in clinical snakebite envenoming. The pharmacists were certified logisticians working in hospital and industrial settings who handle ASV in their line of duties. Each panel member has more than 10 years of working experience. The panel members were asked to review the items in the tool and provide feedback in terms of simple wording and appropriateness to measure ASV knowledge among HCPs.

#### 2.2.2 Content validity

The content validity was ensured based on the recommendation of Polit et al. (2007). To ensure each item in the tool was relevant to measure the ASV knowledge among the HCPs (content validity), a panel of seven experts with qualifications, knowledge, and experience on ASVs was constituted. The panel was requested to review and rate each item in the tool based on a 4-point Likert scale (4-very relevant, 3-relevant, 2-irrelevant, 1-not relevant). The content validity was measured using the content validity index (CVI). The CVI was calculated by dividing the number of participants who rated an item “4” and “3” by the total number of participants. The CVI for the entire scale average (S-CVI/Ave) was determined by taking the average CVI values of items with CVI greater than 0.7. The selection of each item in the tool for inclusion in the final draft was based on the CVI threshold value of greater than 0.7, as recommended by Davis (1992); Polit and Beck (2006). Items with CVI of less than 0.7 were removed from the tool.

### 2.3 Validation study and other psychometric testing

We conducted a validation study and other psychometric tests to assess the tool for construct validity, reliability, difficulty index, and readability. Participants included in this study were the target population for the ASV knowledge assessment tool. The study groups included doctors, pharmacists, nurses, pharmacy technicians, and the general public (≥aged 18 years). Members of the general public were added to assess the tool's ability to discriminate ASV knowledge among groups of people with different health/medical backgrounds.

The sample size was not intended to represent the population of the target participant; it was instead selected only for psychometric validation of the questionnaire (contrast group approach). Based on the available literature, there is no recommended minimum sample size selection for the contrast group approach in psychometric validation studies (pre-testing) of a questionnaire. Previous studies have used different sample sizes in health-related questionnaire validation, ranging from 5 to 75 participants (Wild et al., 2005; Obamiro et al., 2016). To guide the desirable sample size of pre-tests psychometric evaluation of a questionnaire, Perneger et al. (2014) suggested 22–30 participants as an adequate sample size for psychometric validation of health-related tools. Therefore, we adopted the Perneger et al. (2014) recommendation of 30 participants in our validation study.

Participants were recruited through an online survey (via Google Form™). The survey was conducted and reported based on the Checklist for Reporting Results of Internet E-Surveys (CHERRIES) (Eysenbach, 2004). The draft ASV tool was designed in the form of a Google Form. The hyperlink to the online survey was shared with a convenience sample of the target participants via WhatsApp accounts and email addresses. The participants were recruited from six hospitals and pharmacies in rural and urban settings within northern Nigeria. In this survey, the consent was implied by submitting the survey.

#### 2.3.1 Construct validity

Construct validity of the tool was ensured using a contrast group approach based on the recommendation of Polit et al. (2007). The construct validity test was performed to ensure the tool measures the construct (ASV knowledge) among the HCPs. In the contrast group approach, the tool's ability to discriminate knowledge among people with different knowledge and experience in handling ASV was tested. We hypothesized that the ASV knowledge score would be higher among pharmacists compared to doctors, pharmacy technicians, nurses, and members of the general public.

The ASV knowledge of the participants was measured by taking the average percentage scores of correct responses (i.e., dividing the correct scores by the total scores).

#### 2.3.2 Reliability

To ensure the tool reliably measures the ASVs among the HCPs over time, we conducted a test-retest reliability analysis. In selecting an optimal time interval for test-retest reliability, Streiner et al. (2015) recommended that the period be sufficiently short that the attribute of interest does not change due to confounders but long enough that the respondents do not recall the baseline assessment. Gnambs (2014) also found that the administration interval with significantly longer intervals (months) results in lower reliability estimates. Different intervals have been used by researchers to examine temporal consistency in test-retest reliability assessments. Some studies used intervals between two and four weeks (Marx et al., 2003; Sharmila et al., 2013). In situations such as knowledge assessment where changes may not occur daily or even weekly, recall over the four weeks may be more informative (Waltz et al., 2005; Gnambs, 2014). In this study, the test-retest reliability was conducted by administering the tool to the same group of participants at two different times (four weeks’ interval). The reliability was determined by comparing the mean ASV knowledge of the participants for the test-retest survey. Concordant ASV knowledge scores between the two tests indicate excellent reliability (DeVon et al., 2007).

#### 2.3.3 Readability

To ensure the tool is easy to read by most of the target population, it was assessed for readability using the Simple Measure of Gobbledygook (SMOG) grade where a SMOG grade of six and above was considered acceptable (Contreras et al., 1999).

#### 2.3.4 Difficulty index

Items that seem ambiguous to the participants were identified by taking the percentage of correct responses for each item. An item with an ASV knowledge score of below 50% was considered severe and reworded as recommended by UOW (2019).

### 2.4 Statistical analysis

Statistical analyses were conducted and reported based on Statistical Analyses and Methods in the Published Literature (SAMPL) guidelines for basic statistical reporting (Lang and Altman 2014). The analyses were performed using the IBM SPSS Statistics for Windows, Version 22.0. Armonk, NY: IBM Corp. Data were exported from the Google Forms to the SPSS software. Categorical variables were presented as frequency and percentages, while continuous data as mean and standard deviation (SD) or median, the interquartile range for non-normally distributed numerical data. The difference in ASV knowledge among the groups was analysed using the one-way analysis of variance (ANOVA) test, followed by Dunnett's post hoc pairwise comparisons based on the normal distribution of the data. The difference in the mean ASV knowledge for the test and re-test reliability was analysed using paired *t*-test.

## 3 Results

### 3.1 Development

**Fig. 1:**
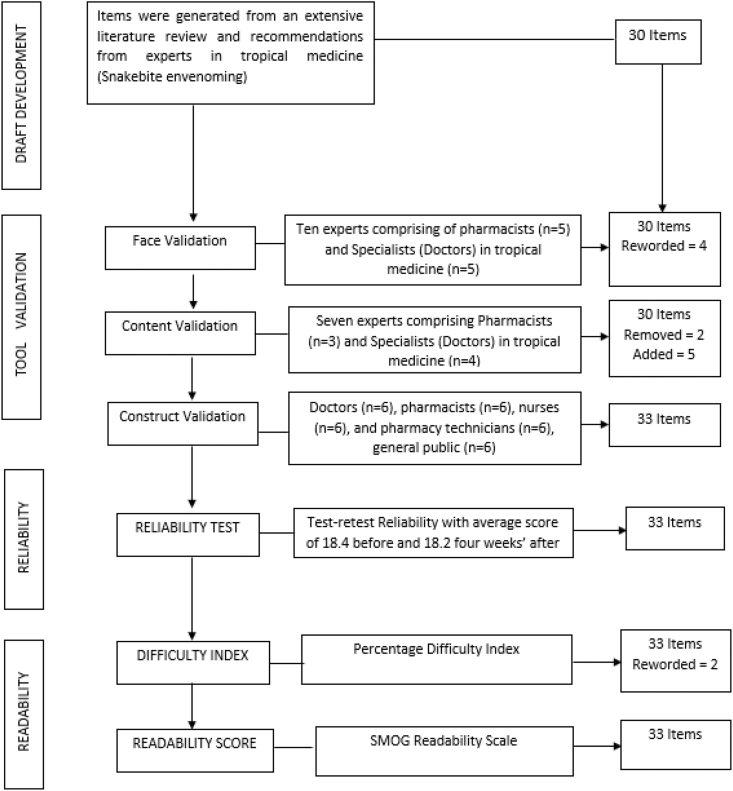
A flowchart of the development and validation process of the antisnake venom knowledge assessment tool (AKAT). SMOG: Simplified Measure of Gobbledygook.

The first draft of the tool consisting of 30 items, was developed from the review of the literature generated and discussion with experts. The flowchart for the development and validation processes is shown in Fig. 1.

#### 3.1.1 Face validity

The draft tool was revised based on the feedback received from a ten-member panel. Most of the comments were about wording and sentence structure. Four items were reworded based on expert recommendations because of ambiguity and grammar.

#### 3.1.2 Content validity

Seven experts reviewed and rated the 30 items in the draft tool. Twenty-six items had I-CVI values of more than 0.78 were retained in the tool on the recommendation of Polit et al. (2007). Four items had an I-CVI of less than 0.78 and deleted from the tool. However, two of those items were retained because they were considered relevant following discussion among the authors.

The expert panel recommended the inclusion of five new items based on clinical relevance. These items were added after a review by the investigators. These items were “Traditional herbs are more efficient than antisnake venom,” “All forms of antisnake venom need to be reconstituted before use,” “the tourniquet should be applied before the administration of antisnake venom,” “are you familiar with snake species in your environment, and the corresponding antisnake venom against them?” and “What is the brand name of the dosage formulation?”

Finally, the S-CVI/Ave of the tool was calculated to be 0.91. The summary of the content validity index determination is shown in Table 1.

**Table 1:** Content validation.

Item No.	Items	I-CVI	Remarks
1	What is the major component of antisnake venom?	0.86	Retained
2	Can antisnake venom be in the following forms?	1.0	Retained
3	What are the standard dosage formulations of antisnake venom?	1.0	Retained
4	Have you ever administered antivenom to snakebite victims?	0.86	Retained
5	What is the appropriate dose of antisnake venom for an adult victim?	1.0	Retained
6	Have you ever been trained on snakebite and antisnake venom?	0.87	Retained
7	What was the medium of training?	0.71	Retained*
8	Monovalent antisnake venom can be used to manage snakebite from two or more snakes	1.0	Retained
9	The appropriate type of antisnake venom for managing snakebite of unknown species is	1.0	Retained
10	The following are major side effects of antisnake venom	0.71	Deleted
11	Treatment for antisnake venom reaction could include	1.0	Retained
12	Antisnake venom contains immunoglobulins	1.0	Retained
13	Antisnake venom is specific to snake species	0.87	Retained
14	Antisnake venom is the only standard treatment for envenoming	1.0	Retained
15	Antisnake venom can cause a severe hypersensitivity reaction	1.0	Retained
16	Antisnake venom is readily available in Nigeria	1.0	Retained
17	Antisnake venom can be administered orally	1.0	Retained
18	Antisnake venom can be administered intravenously	1.0	Retained
19	Antisnake venom can be administered intramuscularly	1.0	Retained
20	Antisnake venom can be administered intradermally	1.0	Retained
21	In the last 24 months, has your facility distributed/stocked antisnake venom?	1.0	Retained
22	What was the brand name of the antisnake venom?	0.87	Retained
23	State the quantity	0.87	Retained
24	What is the type of antisnake venom?	1.0	Retained
25	What is the dosage formulation?	0.71	Retained*
26	What is the name of the antisnake venom?	0.71	Deleted
27	How did you obtain/purchased the antisnake venom?	0.87	Retained
28	What is the average cost of the antisnake venom per vial?	1.0	Retained
29	Where was the antisnake venom stored in your facility?	1.0	Retained
30	In your opinion, what are the appropriate means of transporting antisnake venom?	0.87	Retained

I-CVI, Item-level Content Validity Index; Remarks based on I-CVI threshold of <0.78; * = Reworded and retained based on expert recommendation despite failing to get the I-CVI threshold.

### 3.2 Validation study

A total of 30 participants responded to the survey. Six respondents from each study groups (doctors, pharmacists, nurses, pharmacy technicians, and the general public), responded to the survey. Table 2 demonstrates the percentage scores of the correct responses to the individual items.

**Table 2:** Percentage scores of the correct responses of the number attempted to the individual items.

Item No.	Items	% Correct responses
1	What is the major component of antisnake venom?	67
2	Can antivenom be in the following forms?	63
3	What are the standard dosage formulations of antivenom?	83
4	Have you ever administered antivenom to snakebite victims?	100
5	What is the appropriate dose of antivenom for an adult victim?	53
6	Have you ever been trained on snakebite and antivenom?	100
7	What was the medium of training?	100
8	Monovalent antivenom can be used to manage snakebite from two or more snakes	87
9	The appropriate type of antivenom for managing snakebite of unknown species is	60
10	Treatment for Antivenom reaction could include	53
11	Antivenom contain immunoglobulins	87
12	Antivenom is specific to snake species	77
13	Antivenom is the only standard treatment for envenoming	77
14	Antivenom can have a severe hypersensitivity reaction	80
15	Antivenom is readily available in Nigeria	43
16	Antivenom can be administered orally	80
17	Antivenom can be administered intravenously	87
18	Antivenom can be administered intramuscularly	33
19	Antivenom can be administered intradermally	60
20	Traditional herbs are more efficient than antivenom	83
21	All forms of antivenom need to be reconstituted before use	57
22	The tourniquet should be applied before the administration of antivenom	63
23	Are you familiar with snake species in your environment and the corresponding antivenom against them?	53
24	In the last 24 months, has your facility distributed antivenom?	100
25	What was the brand name of the antivenom?	100
26	State the quantity of the antivenom	100
27	What is the type of antivenom?	90
28	What is the dosage formulation?	73
29	What is the brand name of the dosage formulation?	100
30	How did you obtain/Purchase the antivenom?	100
31	What is the average cost of the antivenom per vial?	100
32	Where was the antivenom stored in your facility?	57
33	In your opinion, what are the appropriate means of transporting antivenom?	63

#### 3.2.1 Construct validity

The results of the one-way ANOVA tests are shown in Table 3. The test indicates a statistically significant difference in ASV knowledge scores among the study participants, thus, valid for construct validity. The pharmacists, pharmacy technicians, doctors, nurses and the general public group scored 86.4%, 68.9%, 64.3%, 56.4% and 36.4%, respectively, (F(d) = 156.92 (4); *p <* 0.001). The post hoc analyses indicate that the ASV knowledge score of the pharmacists was significantly higher than that of the pharmacy technicians, doctors, nurses, and the general public group *p <* 0.001.

**Table 3:** The difference in SAV knowledge among the study groups (n = 30).

Study groups	Mean % of ASV knowledge scores
Pharmacists	86.4
Pharmacy technicians	68.9
Doctors	64.3
Nurses	56.4
General public	36.4

(F(d) = 156.92(4); *p <* 0.001).

#### 3.2.2 Reliability tests

The response rate for the retest reliability analysis was 100%. All the participants responded. The mean ASV knowledge scores of the test-retest reliability were 18.04 for the initial test, and 18.2 four weeks after the test. When the two scores were compared, the results of the paired *t*-test showed no statistically significant difference between the ASV knowledge scores of the participants' test and pre-test, *p* = 0.916 (95%CI -0.14 (-2.85 to 2.57)).

#### 3.2.3 Readability analysis

The SMOG grade of the overall items in the ASV knowledge assessment tool was determined to be grade-level seven. This level implied that, the items in the ASV tool are easy to read and understand by the majority of the target population.

#### 3.2.4 Difficulty index

Two items, *antisnake venom can be administered intramuscularly*, and *antisnake* *venom is readily available in Nigeria,* failed to get the recommended 50% of the correct response. The two items had a correct percentage response of 33% and 43%, respectively, and these items were reworded and retained in the tool based on expert recommendation. The final version of the psychometrically validated ASV Knowledge Assessment Tool (AKAT) is provided as a supplementary material to this article in Appendix 1.

## 4 Discussion

Based on the available literature, this is the first psychometrically validated tool for assessing the ASV knowledge of HCPs. The tool appeared to be valid, reliable, and would enable self-administration by a wide range of HCPs. As a category A neglected tropical disease, it is very paramount to assess the knowledge of HCPs involved in the management of snakebite envenoming. Michael et al. (2018) used a tool for the assessment of first aid and general knowledge of ASV. However, the tool was limited by assessing the general knowledge of SBE among doctors only. Given that ASV is the only scientifically validated therapy for the management of snakebite envenoming, we developed a tool that can assess a broad knowledge of ASV among HCPs such as doctors, pharmacists, nurses, and pharmacy technicians.

All the methods used in this study were based on established methods and guidelines for the development and validation of a tool (Contreras et al., 1999; Tsang et al., 2017). For the face validity, two schools of thought exist in the literature. Some suggest face validity using experts, while others opined using the lay population (Salkind, 2010). In this study, the experts on ASV were used to assess the tool for face validity because we are developing a tool for the assessment of professionals.

For content validity, we used the methods suggested by Polit et al. (2007) because it is easy to understand and interpret (Polit and Beck, 2006). The item level CVI of the items in the tool were within the acceptable range (Contreras et al., 1999). The Scale level (average) CVI of 0.91 was found to be acceptable.

The contrast group approach was used based on a previous recommendation (Tsang et al., 2017). This approach has been applied in previous studies assessing the knowledge of atrial fibrillation (Jatau et al., 2020) and anticoagulants (Obamiro et al., 2016). The tool was able to discriminate knowledge among people from different knowledge backgrounds, thus, ensuring construct validity. The results of the construct validity demonstrate the usefulness of the tool in assessing the ASV knowledge of HCPs.

The two widely used methods for assessing reliability in the literature are stability reliability (using test-retest methods) and equivalence reliability (by measuring internal consistency using Cronbach's alpha) (DeVon et al., 2007). In this study, the test-retest reliability analysis was applied to ensure the tool is reliable over time. There are divergent opinions regarding the best time interval for test-retest reliability assessments. Some studies use a minimum of 6 h after the initial test, while two to four weeks were recommended as acceptable intervals by Waltz et al. (2005). We used the widely acceptable period of four weeks in the present study, and the tool was found to be reliable.

The readability of the tool was assessed using the SMOG formula. This approach was employed because it was considered to be the gold standard for the readability test of health information materials by the National Institute of Health (National Institute of Health, 2018). There was no established literacy level in Nigeria (study setting) for comparison. However, the ease of readability of our tool (SMOG grade level 7) was acceptable based on a previous recommendation (Contreras et al., 1999) and could be easily understood by the target population.

The current study has a public health implication by keying into one of the major goals of the International Society on Toxinology (IST), which include training on the logistics and use of ASV among HCPs, especially those involved in the handling of ASV from production to the end-user (Gutiérrez et al., 2017). The AKAT could be applied in determining the knowledge gaps of ASV among the HCPs. A strategy that could guide the development, implementation, and evaluation of educational interventions (e.g., IST training) on improving ASV knowledge among the HCPs.

The development of this tool has some limitations. The use of online data collection to recruit the participants may limit the sampling of the target population to those with internet access. This inherent shortcoming may have excluded many eligible respondents. Future studies can use both online and face-to-face survey to recruit participants. Secondly, the result of the reliability test is dependent on the study setting and population of the HCPs used in the analysis. Therefore, studies applying the tool in different settings and populations need to test the reliability of the tool before use. Despite these inherent limitations, the study has added important information to the previous body of knowledge of ASV in the literature. Also, the study has provided preliminary data on ASV that could be explored subsequently in future research.

### 4.1 Management implications and adaptability

Antisnake venom is the only specific drug for the treatment of snakebite envenoming. However, there are no local manufacturers of ASVs in Nigeria and other African countries inflicted with the SBE crisis. ASV is being imported from Asia, Europe, and South America. These factors make knowledge of ASV and its logistics vital to HCPs involved in handling it, from the manufacturer, through the rigorous logistic of this delicate and scares drug to the end-user. Because of this, AKAT can be utilized by healthcare facility administrators and policymakers to assess the knowledge of HCPs to improve drug safety, rational use of ASV, and provide educational intervention where necessary. This tool can be directly utilized in Nigeria or adopted by re-conducting a test-retest reliability analysis among a different target population of HCPs. The target users can access AKAT via the website of the journal, scholarly profiles of the authors, and on request from the investigators.

## 5 Conclusion

This study has developed a valid and reliable tool for assessing the knowledge of ASV among HCPs. The tool (AKAT) has demonstrated good psychometrical properties that would enable its application among a wide range of healthcare practitioners.

## Funding

No external funding was used for this study.

## Ethical clearance

The study protocol was reviewed and approved by the Ethics Committee of the College of Health Sciences, Bayero University Kano (BUK/CHS/REC/VII/59). The study was conducted based on the Declarations of Helsinki, 2018.

## Statement of authorship

We declared that this work was conducted by the authors named in this article, and they bore all liabilities relating to the content of this article. All authors have met the criteria for authorship of the Young Pharmacists Scholars (YPS) and International Committee of Editors.

Auwal A. Bala conceived the original idea, developed the theory, and co-wrote the manuscript. Ibrahim Jatau A. developed the study methods and co-wrote the manuscript. Ismaeel Yunusa and Mustapha Mohammed, designed and conducted the formal analysis. Al-Kassim H. Mohammed wrote the introduction, data curation, and co-wrote the manuscript. Abubakar M. Isa. and Wada A. Sadiq, did the literature review, and data curation. Kabiru A. Gulma and Inuwa Bello designed the questionnaire, co-wrote, and edited the final tool. Godpower C. Michael and Sani Malami edited and critically reviewed the manuscript for intellectual content. Basheer A.Z. Chedi gave the supervisory approval and finally revised the manuscript for intellectual content.

## Declaration of competing interest

The authors declare that they have no known competing financial interests or personal relationships that could have appeared to influence the work reported in this paper.
