# Supplementary material for: Development and validation of antisnake venom knowledge assessment tool (AKAT) for healthcare practitioners
Source: Toxicon X. 2020 Dec 3;8:100064. doi: 10.1016/j.toxcx.2020.100064 (PMC7726448; doi:10.1016/j.toxcx.2020.100064)
Supplement: Multimedia component 1 [file mmc1.docx]

**Appendix A**

**QUESTIONNAIRE (TOOL)**

**Section A. Sociodemographic information**

(Tick αand/or insert the correct response)

**1. Gender: □ Male □ Female**

**2. Age**______________

**3. Professional qualification □** *Pharmacist* **□** *Pharm. Tech.* **□** *Nurse* **□** *Doctor*

**4. Additional qualification □** *M.Sc.* **□** *PhD* **□** *Fellowship* **□** *MPH* **□** *Other(s)_________*

**5. Year of Graduation ­­­­­­­­­­­­­­­­_________________________**

**6. Place of Practice□** *Tertiary hospital* **□** *Secondary hospital* **□** *Community pharmacy* **□** *Private* **□** *NGOs*

**7. Location of Practice □** *Urban* **□** *Rural*

**8. State of Practice: □ Bauchi □ Kano □ Niger □ Other** *________________*

**Section B. Knowledge of antisnake venom, dosage, and mode of administration**

***(Tick αthe correct response, unless instructed otherwise)***

**1. The major component of antisnake venom is**

**□** *Carbohydrate* **□** *Protein* **□** *Fat* **□** *Heavy metal*

**2. Antisnake venom can be in the following forms (you can tick more than one answer)**

**□** Monovalent **□** Divalent **□** Tetravalent **□** Polyvalent

**3. What are the common dosage formulations of antisnake venom (you can tick more than one option) □** *Tablets* **□** *Liquid for injection* **□** *Powder for injection* **□** *Topical*

**4. Have you ever administered antisnake venom to snakebite a victim? □ YES □ NO**

**5. What is the appropriate dose of antisnake venom for an adult victim?**

**□** *One vial* **□** *2-3 vials* **□** *Based on patient’s presentation* **□** *Other­­­­­­­­­­­­­(s)_________________*

**6. Have you ever been trained on snakebite management? □ YES □ NO**

**If NO above, skip to question 8**

**7. If YES above, what was the medium of training (you can tick more than one option)**

**□** *Workshop* **□** *Textbooks* **□** *Senior colleague* **□** *Leaflets* **□** *Posters* **□** *At school*

**8. Monovalent antisnake venom can be used to manage snakebite from two or more snake types: □ TRUE □ FALSE**

**9. What is the appropriate type of antisnake venom for managing snakebite of unknown species? □** *Monovalent* **□** *Divalent* **□** *Tetravalent* **□** *Polyvalent*

**10. Treatment for antisnake venom reaction could include: (you can tick more than one option): □** *Adrenaline* **□** *Antihistamines* **□** *Analgesics* **□** *Antihypertensive*

**(Question 11-23, tick the correct response)**

| 11 | **Antisnake venom contain immunoglobulins** | **Yes** | **No** |
| --- | --- | --- | --- |
| 12 | **Antisnake venom is specific to snake specie** |  |  |
| 13 | **Antisnake venom is the only standard treatment for envenoming** |  |  |
| 14 | **Antisnake venom can cause a severe hypersensitivity reaction** |  |  |
| 15 | **Antisnake venom is affordable for snakebite victims in Nigeria** |  |  |
| 16 | **Antisnake venom can be administered orally** |  |  |
| 17 | **Antisnake venom can be administered intravenously** |  |  |
| 18 | **Antisnake venom is preferably administered intramuscularly** |  |  |
| 19 | **Antisnake venom is preferably administered intradermally** |  |  |
| 20 | **Traditional herbs are more efficient than antisnake venom** |  |  |
| 21 | **All forms of antisnake venom need to be reconstituted before use** |  |  |
| 22 | **The tourniquet should be applied before the administration of antisnake venom** |  |  |
| 23 | **Are you familiar with snake species in your environment and the corresponding antisnake venom against them** |  |  |

**Section C. Availability, Cost and Logistics of anti-Snake venom**

***(Tick α and/or insert the correct response)***

**(1) In the last 24 months, has your facility distributed/stocked antisnake venom? □ YES □ NO**

**If NO above, skip to question (10)**

**(2) If YES above, what was the brand name of the antisnake venom? _______________________**

**(3) If YES above, state the quantity______________**

**(4) If YES above, what is the type of the antisnake venom? □**Monovalent **□**Polyvalent **□**Both

**(5) If YES above, what is the dosage formulation? □**Liquid Preparation **□**Powder Preparation

**(6) If YES above, what is the brand name of the antisnake venom**

**□** *EchiTab Plus* **□** *EchiTab G* **□** *Antivipmyn* **□** *PremiumASV* **□** *Other(s) ____________*

(**7) How did you obtain/purchased the antisnake venom**

**□** *Direct from the manufacturer* **□** *From wholesalers/Distributors* **□** *From Retail Pharmacy* **□** *From Government Distribution Agency* **□** *Non-Governmental Organisation (NGOs)*

**(8) What is the average cost of the antisnake venom per vial?**

**□** *Less than 5, 000* **□** *8,000-50,000* **□** *16,000-25,000* **□** *26,000-35, 0000* **□** *≥ 40,000*

**(9) Where was the antisnake venom stored in your facility?**

**□** *Shelf* **□** *Refrigerator* **□** *Room Temperature* **□** *Other* (s)­­­­­­­­­­­­­­________________

**(10) In your opinion, what is the appropriate means of transporting antisnake venom?**

**______________________________________________________________________________________________________________________________________________________**

**Thank you for participating!**
